# Production of Polymeric Membranes Functionalized with Vitreous Humor and Secretome from Dental Pulp Stem Cells for Use in Epithelial Regeneration

**DOI:** 10.1021/acsabm.5c00743

**Published:** 2025-07-15

**Authors:** Wallady da Silva Barroso, Erika Patrícia Chagas Gomes Luz, Lidyane Souto Maciel Marques, Paulo Eduardo da Silva Cavalcante, Francisco Fábio Pereira de Souza, Adriano Lincoln Albuquerque Mattos, Fabia Karine Andrade, André Luís Coelho da Silva, Mariáh Cationi Hirata, Isadora Bosco, Daniel Navarro da Rocha, Renata Francielle Bombaldi de Souza, Fernanda Carla Bombaldi de Souza, José Ricardo Muniz Ferreira, Rodrigo Silveira Vieira

**Affiliations:** † 28121 Federal University of Ceará (UFC), Department of Biochemistry and Molecular Biology, Bloco 907, Fortaleza, Ceará CE 60455-760, Brazil; ‡ 28121 Federal University of Ceará (UFC), Department of Chemical Engineering, Fortaleza, Ceará CE 60455-760, Brazil; § 67838 Embrapa Agroindústria Tropical − CNPAT, Rua Dra Sara Mesquita 2270, Pici, Fortaleza, Ceará CE 60511-110, Brazil; ∥ 28132 R-Crio Stem Cells, Department of Bioengineering, Rua Cumaru 204, Campinas, Sao Paulo 13098-324, Brazil; ⊥ Military Institute of Engineering (IME), Department of Materials Engineering-SE/8, Rio de Janeiro, Rio de Janeiro 22290-270, Brazil

**Keywords:** bacterial cellulose, vitreous humor, biological waste, secretome, wound dressings

## Abstract

This study investigated the development of porous membranes based on a matrix of bacterial cellulose (BC) and sodium alginate (ALG) incorporated with proteins extracted from the tuna vitreous humor and complete conditioned medium (CCM) obtained from the *in vitro* culture of dental pulp stem cells. These bioactive components were integrated into BC-ALG membranes, which were then evaluated for morphology, physicochemical properties, *in vitro* cytotoxicity, and regenerative potential *in vitro*. The membranes exhibited high porosity (72% to 89%), and remarkable swelling capacity reaching up to 3376 ± 0.78% in 72 min, indicating excellent moisture retention essential for wound healing *in vivo*. Cytotoxicity assay showed that none of the formulations were cytotoxic to the tested mammalian cells, confirming their cytocompatibility. Membranes containing CCM showed enhanced cell viability at lower concentrations (500 μg/mL and 250 μg/mL). *In vitro* scratch assays demonstrated that membranes with vitreous humor (VH) and CCM reduced scratch closure time from 18 to 12 h, suggesting their potential for accelerating skin regeneration. This work underscores the promise of using sustainable, waste-derived materials for creating bioactive membranes with applications in tissue engineering and regenerative medicine.

## Graphical abstract

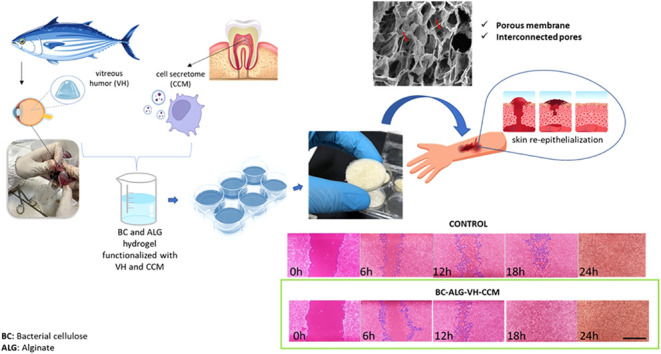

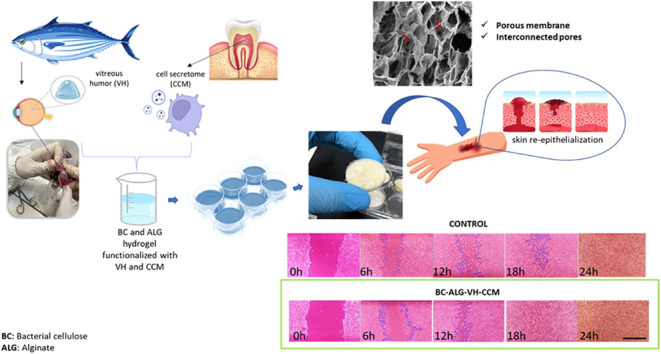

## 1 Introduction

Skin wounds disrupt the organ’s continuity, compromising its integrity to varying degrees and leading to a loss of normal tissue function.^1,2^ This disruption can be caused by physical, chemical, or mechanical trauma or triggered by underlying clinical conditions, which activate the body’s defense mechanisms to repair the damage. Among the most common types of skin wounds are those caused by pressure, mobility restrictions (whether temporary or permanent), vascular insufficiency, trauma, and diabetes-related complications, which are particularly prevalent.^3^

The injury repair process involves sequential stages—hemostasis, inflammation, proliferation, and maturation—regulated by mechanisms crucial to tissue healing, which restores normal architecture and function.^4^ Factors such as age, nutrition, infections, and genetic variations can influence this process, leading to outcomes ranging from normal healing to chronic wounds, which are defined as injuries that remain open for over a month.^5^

Understanding the various types of dressings, their components, and their properties is essential for selecting and applying the appropriate dressing based on the specific clinical needs of each patient.^6^ Traditional dressings, such as gauze, cotton, and tapes, were primarily designed to passively cover wounds, forming barriers against physical and biological agents. These dressings remain commonly used for various injuries, from minor cuts and abrasions to more severe lacerations and burns.

In addition to traditional dressings, modern wound care has introduced bioactive wound dressings. The incorporation of bioactive components capable of modulating the wound microenvironment, combating infections, and stimulating tissue regeneration has been widely explored.^7^ Smart systems, such as injectable, adhesive, and self-healing hydrogels, have been developed to meet the specific demands of complex wounds.^8,9^ These advanced bioengineered materials actively interact with the wound bed to promote healing by maintaining a moist environment, controlling infections, supporting cell proliferation, and stimulating angiogenesis and tissue regeneration.^5,10^ The development and optimization of these advanced dressings represent a significant leap forward in wound care, offering more targeted and effective treatment options than traditional methods.

Given this scenario, the development of new dressings to address chronic wounds using biomaterials and bioactive compounds is essential. Beyond meeting the functional requirements for wound healing, innovation in these advanced dressings can explore novel approaches, such as integrating alternative and sustainable sources of bioactive molecules.^11^ One particularly promising avenue is the utilization of industrial byproducts as bioactive components. Transforming waste materials into valuable resources not only provides a sustainable solution but also reduces production costs, making advanced wound care treatments more accessible. Harnessing these unconventional sources enables the creation of more affordable and effective dressings for chronic wounds, helping to meet the growing demand for innovative therapies in wound management.

Building on the potential of industrial byproducts, proteins isolated from fishing industry waste, such as vitreous humor, present a promising avenue for tissue engineering. The vitreous humor of fish, a transparent and gelatinous substance found in the eye cavity, is rich in bioactive molecules similar to those in mammals, including glycosaminoglycans, proteins, salts, lipids, and other compounds. These components have been shown to enhance biocompatibility and promote cellular proliferation, making them valuable for applications in tissue regeneration.^12^

Scientific evidence suggests that the composition of the vitreous humor varies across different fish species, being influenced by physiological and pathological factors, as well as by specific adaptations to the environment and physiology of each organism, in a manner analogous to what occurs in the human vitreous humor.^13^ Furthermore, recent advances in proteomic analyses have demonstrated that the vitreous humor presents a significantly greater structural and functional complexity than initially assumed. Variations in its composition directly reflect the physiological and pathological state of the ocular tissues, especially the retina and its vascularization.^14^

Among the diverse bioactive molecules used in tissue regeneration, extracellular vesicles (EVs) have emerged as a particularly innovative and effective option. These vesicles, initially identified as subcellular materials derived from platelets in plasma and blood serum,^15^ were first studied for their role in blood clotting. Subsequent research revealed their critical function in cell–cell communication across various cell types.^16^ One particularly potent source of EVs is the secretome a medium enriched with EVs, proteins, and growth factors secreted by cultured cells (also known as conditioned medium). Functionalizing biomaterials with the secretome derived from dental pulp mesenchymal stem cells offers a significant potential for enhancing wound healing and tissue regeneration. By integrating these bioactive compounds, advanced membranes can further support cellular repair and accelerate the regeneration process.

Given the increasing demand for technologically advanced wound dressings this research developed membranes composed of bacterial cellulose and alginate, functionalized with soluble proteins from the vitreous humor (VH) of (Tuna) and conditioned medium (CCM), or the secretome from dental pulp stem cells. These bioactives, sourced from the byproducts of the fishing and pharmaceutical industries, represent a sustainable and innovative approach to wound care. The membranes were evaluated for their morphological and physicochemical properties, *in vitro* cytotoxicity and potential to promote injury regeneration in mammalian epithelial cell lines. These assessments aim to demonstrate the feasibility of using these bioactive-functionalized membranes in treating human skin wounds, highlighting their potential as effective, eco-friendly alternatives for advanced wound healing.

## 2 Material and Methods

### 2.1 Chemicals and Materials

Potassium carbonate (K_2_CO_3_, Dynamic), agar–agar (bacteriological, Dynamic), HS broth: citric acid (C_6_H_8_O_7_, Neon), yeast extract (NCM0218A-HX0161-00055, Neogen), d-(+)-glucose (C_6_H_12_O_6_, Neon), dibasic sodium phosphate (Na_2_HPO_4_, Dynamics), and peptone (bacteriological, Kasvi).

### 2.2 Collect of Vitreous Humor from Tuna, Extraction, and Characterization of Vitreous Humor Proteins (VH)

The eyeballs of (Tuna) were collected from a local fish processing industry. Fresh tuna eyeballs were collected using the aid of surgical instruments, placed into 150 mM NaCl sterile solution, and immediately transported to the laboratory under refrigeration. In the laboratory, tuna eyes were immersed in 70% alcohol solution for 5 min to minimize microbial contamination. After, the alcohol solution was removed and the eyes were frozen in an ultrafreezer to facilitate removal of the vitreous humor (VH). For VH extraction, the frozen eyes were immobilized on a Styrofoam plate covered with plastic film using pins for stabilization. Then, a cross-shaped incision was made in the anterior region of the eyeball, near the insertion point of the optic nerve, using a blade and scalpel to expose the VH. Additionally, the lyophilized VH were previously characterized by scanning electron microscopy (SEM). For this analysis, the samples were fixed on brass stubs with carbon tapes and coated with a thin layer of silver (20 nm) using a sputter applicator (K650, Emitech, France). The samples were observed under a Quanta 450 FEG scanning electron microscope (FEI Company, Hillsboro, Oregon, USA).

For protein extraction, VH was mixed at a 1:2 w/v ratio in 150 mM NaCl sterile solution, placed into a centrifuge tube (50 mL) containing glass beads, vortex for 1 min, and centrifuged at 25,500*g* for 15 min at 4 °C. The supernatant was collected and the precipitate underwent a re-extraction process following the steps described previously. The supernatants were filtered through a 0.22 μm filter, frozen in a −20 °C freezer and lyophilized to facilitate storage and preservation.

The protein extracts generated were analyzed by polyacrylamide gel electrophoresis under reducing conditions according to the methodology of Laemmli,^17^ with modifications. The analysis utilized a stacking gel with 3.5% acrylamide in 0.5 M Tris-HCl buffer (pH 6.8) and a separating gel with 12.5% acrylamide in 0.5 M Tris-HCl buffer (pH 8.8) both containing 1% SDS. Electrophoretic mobility was performed at 25 mA and an initial voltage of 100 V. Samples were prepared in 62.5 mM Tris-HCl buffer (pH 8.3) containing 1% SDS, 0.1% β-mercaptoethanol, sucrose and bromophenol blue 1%. The samples were then heated to 100 °C for 10 min and centrifuged at 1,000*g* for 5 min at room temperature. The protein bands were stained with 0.05% Coomassie Brilliant Blue R-250, prepared in a solution of methanol: acetic acid: water (1:3.5:8, v/v/v). The Gel Filtration Markers Kit for Protein Molecular Weights 12,000–200,000 Da was used as a molecular marker.

#### 2.2.1 Cytotoxicity of VH Protein Extract

The cytotoxicity against fibroblasts (L929) of the protein extract from tuna VH was evaluated by a direct method, following the methodology described by the regulatory standard ISO10993-12 ^18^ in which cell viability is measured after exposing the cells to the sample for 24 and 48 h. The lyophilized protein extracted from Tuna VH was dissolved in the medium used for cell cultivation at concentrations of 1 and 0.1 mg/mL. The reagent used to measure the metabolic activity/viability of the cells at the end of the analysis period was Resazurin. Three independent experiments were conducted to evaluate the cytotoxicity of the sample at different concentrations, with three replicates per condition in each assay.

### 2.3 Obtainment and Characterization of Complete Conditioned Medium (CCM) from Dental Pulp Stem Cells

Dental pulp stem cells (DPSCs) from a 6-year-old female donor (ÍHFCK) were obtained with informed consent after the responsible guardian was duly instructed about the surgical protocol. Following the signature of the informed consent form, the dental pulp was collected under aseptic conditions and processed for culture in standard laboratory environments to produce complete conditioned medium (CCM). This procedure was approved by the National Commission for Research Ethics under approval number 4.348.204.

Briefly, DPSCs were seeded at an initial density of 10,000 cells/cm^2^ in 3.85 cm^2^ culture wells (P0) and maintained in a defined growth medium (HM, 1001537, exp. 02/24/2024, Millipore), supplemented with ascorbic acid (0143/23, exp. 09/15/2023), l-glutamine (RNBK5868, exp. 10/2023, Sigma), penicillin (40174185, exp. 05/22/2024, Gibco), and human serum (SLK7753, exp. 08/31/2026; SLCL9018, exp. 04/30/2027, Sigma), at 37 °C in a humidified atmosphere containing 5% CO_2_.

Cells were grown to approximately 90% confluence before passaging. For passaging, cells were detached from the culture surface using TrypLE (2522531, exp. 11/30/2024, Gibco), and the enzymatic reaction was neutralized with complete growth medium. The cell suspension was centrifuged, counted, resuspended in fresh medium, and reseeded at an initial density of 5,000 cells/cm^2^ in 25 cm^2^ culture flasks.

Conditioned medium was collected between passages P0 and P1. The harvested medium was centrifuged to remove cellular debris, aliquoted, frozen for 24 h at −20 °C in a freezer (Analytical, model HOTA 20FL), and lyophilized (Christ, model Alpha 1-2) for 48 h under a vacuum lower than or equal to 0.040 mbar for long-term storage and subsequent characterization by atomic force microscope, particle size and zeta potential. Its direct cytotoxicity *in vitro* was evaluated against L929 cell lines.

To analyze the size of the CCM particles present in the samples used, Atomic Force Microscopy analysis was performed. For this, a 2 mg/mL solution of CCM in PBS was prepared, then centrifuged at 5000*g* for 5 min and filtered through a 0.22 μm membrane. The CCMs were fixed in 2% glutaraldehyde, washed with PBS-Tween and centrifuged again. The pellet was resuspended in ultrapure water and immediately applied to glass slides for microscopy. The samples were dried at room temperature for 1 h and then dehydrated in a gradient of 50, 70, 90 and 100% ethanol. An MFP-3D-BIO microscope (Asylum Reasearch) was used to scan the samples fixed in tapping mode. The cantilever used has a spring constant of 40 N/m. Images were obtained at a resolution of 256 × 256 pixels, with a scanning rate of 0.3 to 0.75 Hz. In addition to this analysis, particle size and zeta potential were performed.

The average size, distribution of CCM particles and zeta potential were determined by Dynamic Light Scattering (DLS), using the Zetasizer, Nano Series, Malvern brand, model ZEN 3600, with a red light beam and wavelength of 633 nm. To perform this analysis, 1 mL of the sample was removed and stored in specific cuvettes for reading, at 25 °C.

The cytotoxicity against fibroblasts (L929) of the CCM was evaluated by a direct method, following the methodology described by the regulatory standard ISO10993-12 ^18^ in which cell viability is measured after exposing the cells to the sample for 24 and 48 h. The lyophilized CCM was dissolved in the medium used for cell cultivation at concentrations of 1000, 500, 250, and 125 μg/mL. The reagent used to measure the metabolic activity/viability of the cells at the end of the analysis period was Resazurin.

### 2.4 Production, Oxidation, and Deconstruction of Bacterial Cellulose

The cultivation of ATCC 53582 strains and obtainment of bacterial cellulose (BC) membranes were carried out according to the methodology described by Hestrin and Schramm.^19^ After incubation, the BC formed was washed in running water and purified. BC was immersed in distilled water for purification and heated to 80 °C for 1 h. This procedure was performed twice to remove excess culture medium and microbial content. To remove the bacteria and the culture medium altogether, the BC was treated twice with a 0.3 mol/L K_2_CO_3_ solution at 80 °C for 1 h. After purification, the BC were washed with distilled water (25 °C) until they reached neutral pH.

The oxidation of BC was based on the methodology described by Vasconcelos et al.^20^ The purified BC membranes were pretreated by immersion in a KCl-HCl solution pH 1.0 (BC/KCl-HCl – 0.356 g/50 mL) for 24 h and were subsequently added to the reaction system containing sodium periodate (NaIO4) dissolved in KCl-HCl pH 1.0 (BC/NaIO_4_ – 1.0 g/1.5 g). The system was incubated in an orbital shaker (SOLAB – SL 222) at 55 °C, 125 rpm for 6 h in the absence of light. To stop the oxidation reaction, 2.5 mL of ethylene glycol were added to the system, which was incubated for 1 h at 25 °C. Ethylene glycol acts by decomposing the remaining periodate in the solution. The oxidized bacterial cellulose membranes (OBCs) were washed with deionized water (25 °C) until they reached neutral pH. Oxidized CB was used in the study to produce a degradable membrane in physiological fluids, enhancing its use.

The bacterial cellulose membranes were crushed by an electric processor to break the fibers and obtain a homogeneous mixture with the addition of water and carboxymethyl cellulose (CMC) in the following proportions: wet BC/H_2_O/CMC = 180 g/700 mL/3.6 g. The same process was performed to deconstruct the OBC membranes following the proportion wet OBC/H_2_O/CMC = 165 g/356 mL/2.12 g.

### 2.5 Production of Membranes

To produce the membranes, a reinforcement of 2% (w/v) sodium alginate (ALG) and 10 mg/mL VH from Tuna were added per mL of deconstructed OBC for functionalization. The blends were poured into molds (24-well plates or Petri dishes) to which a vacuum of 200 μHg was applied for 30 min to remove any air bubbles present, frozen at −20 °C for a period of 16 h, lyophilized until the material was dehydrated, cross-linked with 0.5 M CaCl2, frozen again at – 20 °C for a period of 16 h (overnight) and lyophilized until the material was dehydrated. Then, 2 mg/mL of the conditioned medium was dissolved in PBS buffer and centrifuged at 5,000 rpm to remove insoluble components and filtered through a 0.22 μm filter, leaving only 0.5 mg/mL of CCM (determined by Bradford assay) to be adsorbed by the lyophilized BC-ALG-VH matrices. The entire process was conducted under sterile conditions. Nonfunctionalized matrices are those without the addition of VH, for comparative purposes. In Table 1, the tested formulations are described.

**Table 1:** Membranes Formulations Based on Bacterial Cellulose Deconstructed and Alginate Functionalized with Fish Vitreous Humor and CMC^a^

Membrane	Bacterial cellulose (w/v)	CMC (w/v)	Alginate (w/v)	VH _Tuna_ (mg/mL)	CCM (mg/mL)
BC-ALG-CCM	26%	0.2%	2%	0	0.5
OBC-ALG-CCM	46%	0.2%	2%	0	0.5
BC-ALG-VH-CCM	26%	0.2%	2%	10	0.5
OBC-ALG-VH-CCM	46%	0.2%	2%	10	0.5

a BC—Bacterial cellulose; OBC—oxidized bacterial cellulose; VH—vitreous humor; CCM—conditioned medium.

### 2.6 Membranes Characterizations

#### 2.6.1 Morphological and Physicochemical Characterization

The study characterized the morphological and physicochemical properties of the developed formulations by scanning electron microscopy (SEM), swelling, porosity, exudate absorption, contact angle and *in vitro* cytotoxicity against keratinocytes (HaCaT). In addition, tests were performed to understand the protein release profile and *in vitro* healing assay (Scratch). For morphological characterization by SEM, the membranes were fixed to brass stubs with carbon tapes and covered with a thin layer of silver (20 nm) using a sputter applicator (K650, Emitech, France), and were observed under a Quanta 450 FEG scanning electron microscope (FEI Company, Hillsboro, Oregon, USA). The images obtained were processed using ImageJ software (version – 1.54f).

The swelling behavior of the membranes was evaluated following the methodology described by Liu and collaborators.^21^ The freeze-dried membranes were cut into circles with an area of approximately 1 cm^2^ and immersed in 10 mL of distilled water (pH 7.0) at 25 °C for 72 min. Excess water was removed with filter paper (Quantity, 8 μm) and the samples were weighed. The degree of swelling was calculated according to eq 1:

1 $$SD\;(\%)=\frac{m_{wet}-m_{dry}}{m_{dry}}\times 100$$

SD is the swelling degree; *m*_wet_ is the mass of the swollen sample after the time interval established for weighing the sample and *m*_dry_ is the dry sample mass before the start of the experiment.^21^ All tests were performed in triplicate.

The porosity measurement (%) of the membranes was determined using the methodology described by Zeng and Ruckenstein.^22^ The membranes previously swollen in distilled water were weighed and then lyophilized. The resulting mass of the freeze-dried matrix was also measured. Based on the volume of water contained in the material, the porosity (%) was calculated using eqs 2 and 3:

2 $$\mathrm{Porosity}\;(\%)=\left(\frac{m_{wet}-m_{dry}}{d_{water}}\right)\times \left(\frac{100}{V_{wet}}\right)$$

where the porosity (%) of the membrane is given by the variables; *m*_wet_, which is the wet mass of each matrix measured after each period; *m*_dry_ is given by the mass of the freeze-dried matrices measured at the beginning of the process; *d*_water_ is the density of pure water at the temperature at which the analysis was carried out (25 °C); *V*_wet_ is described in eq 3

3 $$V_{wet}=\frac{\pi \times D^{2}\times h}{4}$$

where the variables give *D* is the diameter of the matrix and *h* is the height.

The exudate absorption capacity of aqueous solutions was determined using a gravimetric method, by means of the degree of swelling of the membranes in saline solution (0.9% w/v NaCl, pH 5.5). The saline solution is usually applied in the cleaning and hydration of lesions. The samples were previously freeze-dried and their initial weight (*m*_dry_) was measured. Then, the membranes were immersed in 10 mL of the aforementioned solution for 24 h at 37 °C. After this time, the excess liquid was removed using filter paper. Finally, the samples were weighed on an analytical balance to determine the final wet masses (*m*_wet_). The absorption capacity, in percentage, of each solution was calculated using eq 4.

4 $$\mathrm{Exudate}\;\mathrm{absorption}\;(\%)=\frac{\left(m_{\mathrm{wet}}-m_{\mathrm{dry}}\right)}{m_{\mathrm{dry}}}\times 100$$

The contact angle was determined using an optical contact meter (GBX Instrumentation Specifique), in which a drop was deposited on the surface of the membranes. The 2 × 2 cm samples were fixed to a glass support in order to capture the image using a Nikon Pixe Link camera at the moment the drop touched the surface, as well as to measure the angle. The measurements were performed in triplicate.

#### 2.6.2 Protein Releasing Assay

The protein releasing assay was performed to evaluate the capacity of membranes to release to deliver bioactive compounds from VH and CCM. For this purpose, membrane samples (1 cm^2^ each) were immersed in PBS buffer and incubated at 37 °C for periods of 1, 2, and 3 days. After each time point, the extracts were collected, and the total concentration of released proteins – originating from both VH and CCM was determined using the Bradford colorimetric assay. Protein quantification was based on a standard curve generated with bovine serum albumin (BSA). All measurements were performed in triplicate.

#### 2.6.3 In Vitro Cytotoxicity

The cytotoxicity of the materials was evaluated by an indirect method, following the methodology established by regulatory standard ISO10993-12,^18^ in which cell viability is measured after exposing the cells to the sample extracts. To prepare the extracts, the samples were initially sectioned into a circular shape with an approximate area of 1 cm^2^/mL de PBS (0.022 g dry mass) and sterilized by exposure to UV light for 20 min on each side of the disk in a laminar flow chamber. The sterile samples of each matrix were placed in 24-well plates with 1 mL of culture medium per well, part of them with DMEM supplemented (10% FBS and 1% penicillin-streptomycin) and incubated at 37 °C for 24 h (5% CO_2_ and 95% humidity). After the incubation period, the extracts obtained were collected and stored in Falcon conical tubes, taking care not to collect parts of the membranes as well. To perform the assay, adult human keratinocytes (HaCaT) were seeded in DMEM medium supplemented (10% FBS and 1% penicillin-streptomycin) in 96-well plates at a density of 6 × 10^3^ cells/well, followed by incubation at 37 °C (5% CO_2_ and 95% humidity) for 24 h. After this period, the culture medium present in the wells was replaced by 100 μL of the previously prepared extracts and the plates were again incubated at 37 °C for a period of 24 and 48 h (5% CO2 and 95% humidity). After the incubation period, the extracts were removed, the wells were washed with PBS buffer solution (pH 7.4) and 120 μL of culture medium containing 10% (final concentration of 2.5 mg/L) of the resazurin solution were added to each well. The plates were again incubated at 37 °C for 4 h (5% CO_2_ and 95% humidity) and after this period, 100 μL of the product of metabolization by the cells of resazurin was transferred to a new 96-well plate. The plate was read in a microplate reader (SpectraMax i3x, Molecular Device, Sunnyvale, USA), in fluorescence mode (λ excitation = 560 nm and λ emission = 590 nm). As a negative control of the assay, the cells were exposed only to the culture medium and as a positive control, the cells were exposed to a solution of 40% DMSO diluted in DMEM. The percentage of metabolically active cells was calculated using eq 5:

5 $$CV\;(\%)=\frac{F_{\mathrm{sample}}}{F_{\mathrm{control}}}$$

We have that represents cell viability, represents the fluorescence corresponding to the well where the cells were cultured in the presence of the sample extract and represents the fluorescence corresponding to the well where the cells were cultured only in the presence of their respective culture media. The tests were performed in triplicate (*n* = 3), in order to obtain better representativeness of the results.

#### 2.6.4 In Vitro Scratch Assay

The effect of membrane extracts containing VH and CCM on cell migration was assessed using a scratch assay with L-929 fibroblasts. The extracts were prepared using the same protocol described in the previous section (1 cm^2^ membrane sample, exposed to 1 mL PBS, for 24 h). Fibroblasts were seeded in 24-well plates and incubated under the specified conditions until they reached confluence. A sterile 200 μL pipet tip created a scratch across the confluent cell monolayer. Detached cells were removed by washing with PBS, and 1 mL of supplemented DMEM was added to each well. Horizontal reference lines were drawn at the bottom of the plates using a fine pen to assist with alignment during image acquisition. Following this, the plates were transferred to an inverted microscope. Areas of interest were observed under a Nikon microscope using a 10× objective and images were captured every 6 h for 24 h. The section and total section coverage areas were analyzed using a plugin for ImageJ software.^23^ The percentage of wound closure was calculated according to eq 6:

6 $$\mathrm{Scratch}\;\mathrm{closing}\;\%=\left(\frac{A_{t=0}-A_{t=\Delta t}}{A_{t=0}}\right)\times 100\%$$

*A*(*t* = 0) represent the initial area of the wound and *A*(*t* = Δ*t*) represent the area of the wound after *n* hours from the initial incision, both measured in μm^2^. The images were analyzed using the plugin with the following parameters: Variance window radius: 15, threshold value: 100, percentage of saturated pixels: 0.01, global set scale: Yes, Is the scratch diagonal?: No. The wound closure data will undergo regression analysis to identify the best mathematical model representing the results. Linear, quadratic, and cubic regressions were performed, with the regression model chosen based on the coefficient of determination (*R* ^2^) closest to 1.0. The data were statistically analyzed using a two-way ANOVA with Tukey’s multiple comparisons test, performed with GraphPad Prism software (GraphPad Prism 9.4.0.673, GraphPad Software LLC, Dotmatics, Boston, MA, USA). A significance level of *p* < 0.05 was considered statistically significant.

### 2.7 Statistical Analysis

The data obtained were statistically analyzed using the 2-way ANOVA test with Tukey’s multiple comparisons test, with the aid of the GraphPad Prism program (GraphPad Prism 9.4.0.673 - GraphPad Software LLC—Dotmatics, Boston, MA, USA). The significance level adopted was *p* < 0.05.

## 3 Results and Discussion

### 3.1 Extraction and Characterization of Proteins from VH from Tuna

Figure 1A shows the manual extraction procedure of the vitreous humor (VH) from tuna eyes, revealing the gelatinous structure that occupies approximately 80% of the eyeball volume. Figure 1B illustrates the separation of VH from other intraocular components. After removal of the aqueous humor, the crystalline lens and vitreous humor were isolated. The dark liquid present corresponds to residual aqueous humor not fully aspirated during dissection. Protein extraction from VH was carried out at 1:2 (w/v) ratio using 150 mM NaCl as extraction buffer. The soluble protein extract was then sterilized using a 0.22 μm filter and lyophilized for storage. The final pellet, corresponding to the nonsoluble fraction, was also lyophilized and partially resuspended. Then, the protein extracts were evaluated by SDS electrophoresis as shown in Figure 1C.

**Figure 1:**
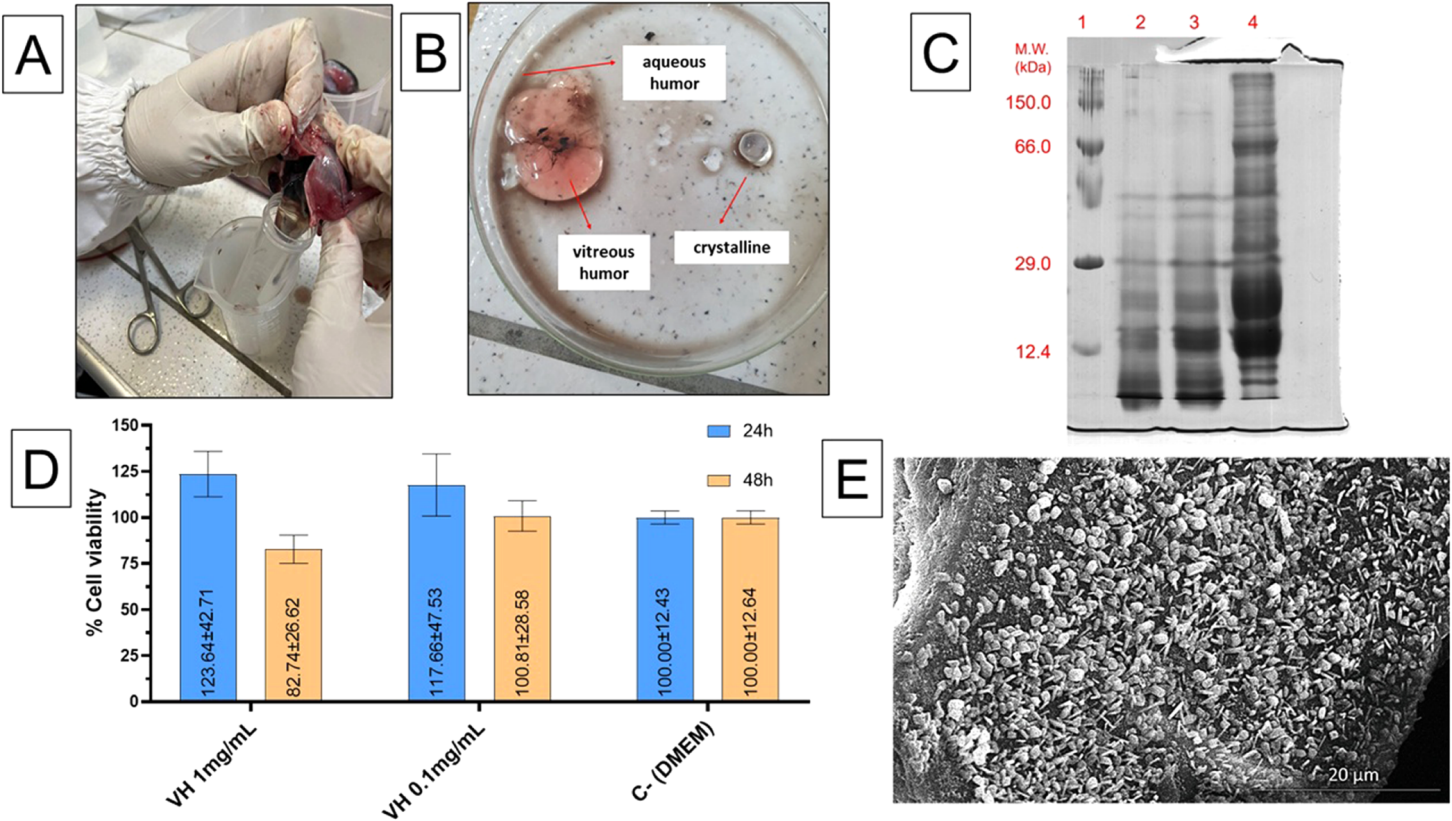
(A) Manual extraction process of vitreous humor (VH) from tuna eyes. (B) Separation of VH and crystalline lens after aspiration and removal of aqueous humor, with some residual dark aqueous liquid visible. (C) SDS-PAGE of VH protein extracts under reducing conditions. Lane 1: molecular weight marker; Lane 2: crude VH extract (unfiltered); Lane 3: filtered VH extract (soluble protein fraction); Lane 4: nonsoluble fraction obtained from the pellet after two extraction cycles, lyophilized and partially resuspended in SDS buffer. (D) Cell viability of L-929 fibroblasts after 24 and 48 h exposure to VH at 0.1 and 1.0 mg/mL. Three independent experiments were conducted to evaluate the cytotoxicity of the sample at different concentrations, with three replicates per condition in each assay. Analysis of statistical difference intergroup (VH 1 and 5 mg/mL v/s control C−) was conducted using two-way ANOVA followed by Tukey’s post hoc test, and values were considered significant if *p* < 0.05. (E) Scanning electron micrograph of lyophilized VH showing irregular aggregates at 5000× magnification.

Figure 1C presents the SDS-PAGE profile of VH protein extracts at different stages of processing. Lane 1 corresponds to the molecular weight marker; Lane 2 to the crude (unfiltered) VH extract; Lane 3 to the filtered VH extract, representing the soluble protein fraction; and Lane 4 to the nonsoluble fraction, which was obtained from the final pellet after two extraction cycles, lyophilized, and subsequently resuspended for electrophoretic analysis. The presence of distinct bands in Lane 4 indicates that at least part of the aggregated or precipitated proteins could be recovered after resuspension, denaturation, and heating. The band intensity observed in Lane 3 was greater than in Lane 2, which may be explained by the removal of insoluble material and interfering substances during filtration. This likely led to improved protein migration and sharper band definition rather than an actual increase in protein content.

Figure 1D shows the results of the cytotoxicity assay performed on L-929 fibroblasts exposed to VH at concentrations of 0.1 and 1.0 mg/mL for 24 and 48 h. Three independent experiments were conducted to evaluate the cytotoxicity of the sample at different concentrations, with three replicates per condition in each assay. After 24 h, cell viability remained at or above 100% across all conditions, suggesting no cytotoxic effects at the tested concentrations. At 48 h, a modest reduction in viability was observed at 1.0 mg/mL (82.7%), but this decrease was not statistically significant compared to the negative control (C−), with a p-value of 0.798 (two-way ANOVA followed by Tukey’s post hoc test). These results support the biocompatibility of VH extracts under the evaluated conditions.

Figure 1E displays the scanning electron microscopy (SEM) image of the lyophilized VH extract. The micrograph reveals heterogeneous aggregates with irregular morphology and varying sizes, which reflect the structural complexity of the VH material. These features enhance the efficiency of the extraction and lyophilization steps, highlighting the diverse physical characteristics of the extracted components.

### 3.2 Characterization of Complete Conditioned Medium (CCM) Obtained from Dental Pulp Stem Cells

Complete conditioned medium (CCM) consists of the secretome of dental pulp mesenchymal stem cells (DPSCs) cultured *in vitro*. This secretome is a complex mixture of bioactive molecules secreted by the cells into the culture medium, including proteins, cytokines, growth factors, and extracellular vesicles (EVs).^24^ To visualize the morphology of particles, present in the CCM, atomic force microscopy (AFM) was performed on a glass substrate. Figure 2A shows spherical nanoscale structures with diameters of approximately 150 nm, consistent with the size range of small EVs (e.g., exosomes), and aggregates of ∼420 nm, likely representing vesicle clusters or other macromolecular assemblies.^25,26^

**Figure 2:**
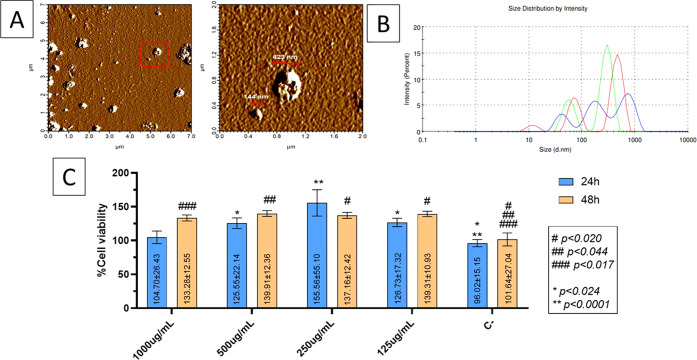
(A) Atomic force microscopy (AFM) images of particles in CCM. Left: overview of the surface. Right: magnified region showing spherical nanostructures with approximate diameters of 144–423 nm. (B) Particle size distribution profile of CCM obtained by dynamic light scattering (DLS) performed in triplicate. (C) Cell viability of L-929 fibroblasts exposed to various concentrations of CCM for 24 and 48 h. The assay was conducted in triplicate (*n* = 3). Two-way ANOVA followed by Tukey’s post hoc test was used for statistical analysis. Asterisks indicate significance vs control at 24 h (*p < 0.024; **p < 0.0001); hashes indicate significance vs control at 48 h (#*p* < 0.020; ##*p* < 0.044; ###*p* < 0.017).

To further characterize the CCM, dynamic light scattering (DLS) and zeta potential analysis were performed to assess particle size distribution and colloidal stability. As shown in Table 2, the mean hydrodynamic diameter was 283.5 nm, with a polydispersity index (PDI) of 0.73, indicating a wide size distribution and low homogeneity. The zeta potential was measured at −18.3 mV, which suggests moderate colloidal stability in suspension. For optimal long-term dispersion, values below −30 mV are typically expected.^27,28^

**Table 2:** Hydrodynamic Diameter (nm), Polydispersity Index (PDI), and Zeta Potential (mV) of CCM in Aqueous Suspension at pH 6, as Measured by Dynamic Light Scattering (DLS) and Zeta Potential Analysis

Parameters	CCM in suspension pH 6
Hydrodynamic diameter (size-nm)	283.5
Polydispersion index (PDI)	0.73
Zeta potential (mV)	–18.3

The particle size distribution curve obtained from dynamic light scattering (DLS) measurements is shown in Figure 2B. The profile confirms the presence of a polydisperse population, with a predominant peak near 280 nm and broader distribution consistent with the observed polydispersity index. These data are consistent with the presence of extracellular vesicles and protein aggregates in the CCM and support the values presented in Table 2.

Although specific molecular content was not profiled in this study, previous reports indicate that CCM derived from DPSCs typically contains growth factors such as VEGF, TGF-β1, HGF, and IGF-1, as well as EVs carrying regulatory microRNAs, all of which have been implicated in promoting cellular proliferation, angiogenesis, and tissue regeneration.^29^

The *in vitro* cytotoxicity of lyophilized CCM was evaluated in L929 fibroblasts to determine a suitable working concentration for incorporation into the membranes. As shown in Figure 2C, all tested concentrations (125–1000 μg/mL) maintained cell viability above 100% after 24 h. At 48 h, the best results were observed at 125 μg/mL and 500 μg/mL, with statistically significant increases in viability. These results show that, in addition to being noncytotoxic, the materials/treatments positively modulated cell proliferation compared to the negative control. Based on these findings, 500 μg/mL was selected as the working concentration due to its consistent performance at both 24 and 48 h, and its suitability for downstream incorporation into membranes, where slightly higher concentrations may help compensate for processing losses and ensure effective bioactivity distribution throughout the biomaterial.

### 3.3 Membrane Synthesis and Characterization

Four membrane formulations were synthesized by combining bacterial cellulose (BC) or oxidized bacterial cellulose (OBC) with alginate (ALG), and functionalized with either VH, CCM, or both bioactives (see Table 1 for composition details).

Table 3 summarizes the physicochemical properties of the membranes, including swelling capacity, exudate absorption, porosity, and contact angle all of which are critical for assessing their potential as wound dressings.^30–33^ The effectiveness of a dressing depends not only on its biocompatibility but also on how it interacts with fluids in the wound environment. These properties influence the ability to retain moisture, absorb exudate, allow gas exchange, and promote favorable interactions at the wound surface.

**Table 3:** Summary of Swelling, Exudate Absorption, Porosity, and Contact Angle of the Synthesized Membranes, Relevant to Their Performance as Wound Dressings

Characteristic	BC-ALG-CCM	OBC-ALG-CCM	BC-ALG-VH-CCM	OBC-ALG-VH-CCM
Swelling (with 72 min)	3119 ± 0.77%	3496 ± 0.88%	2982 ± 0.75%	3376 ± 0.78%
Exudate absorption (after 24 h)	97 ± 0.36%	97 ± 0.55%	97 ± 0.40%	97 ± 0.35%
Porosity	76 ± 0.53%	84 ± 0.60%	72 ± 0.46%	89 ± 0.51%
Contact angle (regime)	52.5° hydrophilic	64.3° hydrophilic	46.7° hydrophilic	49.8° hydrophilic

All formulations demonstrated high swelling capacity, with values ranging from 2982% to 3496% after 72 min. The ability to absorb and retain exudate helps maintain a balanced moist environment, which is known to support tissue repair and re-epithelialization. Notably, OBC-ALG-CCM showed the highest swelling, which may reflect enhanced fluid uptake due to increased surface area or structural porosity, while BC-ALG-VH-CCM showed the lowest, possibly due to stronger intermolecular interactions between VH proteins and the polymeric matrix. These interactions may lead to a denser network structure with reduced pore size and lower availability of hydrophilic groups for water absorption. Additionally, the presence of VH may promote physical cross-linking or hydrogen bonding within the matrix, restricting water diffusion and swelling capacity.

Exudate absorption remained stable across all formulations, reaching approximately 97% after 24 h, a level considered ideal for highly exudative wounds. This property ensures that the dressing can manage large volumes of wound fluid without becoming saturated too quickly, preventing periwound maceration and reducing the risk of infection. The low standard deviations observed suggest consistent performance across replicates.

Porosity values ranged from 72% (BC-ALG-VH-CCM) to 89% (OBC-ALG-VH-CCM). Porosity affects not only fluid absorption but also oxygen and vapor permeability, both of which are important for tissue viability and granulation. The lower porosity in VH-containing membranes may indicate partial occlusion of pores by bioactive incorporation, while the OBC matrices retained higher porosity, potentially due to less collapse during gelation and drying. This phenomenon can be attributed to the interaction between VH proteins and the polymeric chains during membrane formation. VH is rich in proteins with functional groups capable of establishing hydrogen bonds and electrostatic interactions with alginate and bacterial cellulose. These interactions may lead to tighter packing of polymer chains, partial filling of the pore spaces, and structural densification.

The contact angle values were all below 90°, indicating that all membranes are hydrophilic, which is advantageous for wound applications. OBC-ALG-CCM had the highest angle (64.3°), suggesting slightly reduced wettability compared to BC-ALG-VH-CCM (46.7°). This variation may reflect differences in surface chemistry resulting from oxidation or bioactive loading. Hydrophilic surfaces promote better fluid spread and enhance the interface between the dressing and wound bed, supporting exudate distribution and preventing pooling.

Together, these results demonstrate that the synthesized membranes meet key performance criteria for wound healing: rapid swelling, high fluid absorption, optimal porosity, and favorable surface wettability. Subtle differences among formulations may reflect the influence of OBC modification and bioactive incorporation, and these factors should be considered when selecting materials for specific wound types or healing phases.

Figure 3 summarizes the evaluation of the synthesized membranes. Figure 3A shows representative images of the four membrane formulations, all exhibiting a uniform and spongy appearance typical of polymeric porous structures.

**Figure 3:**
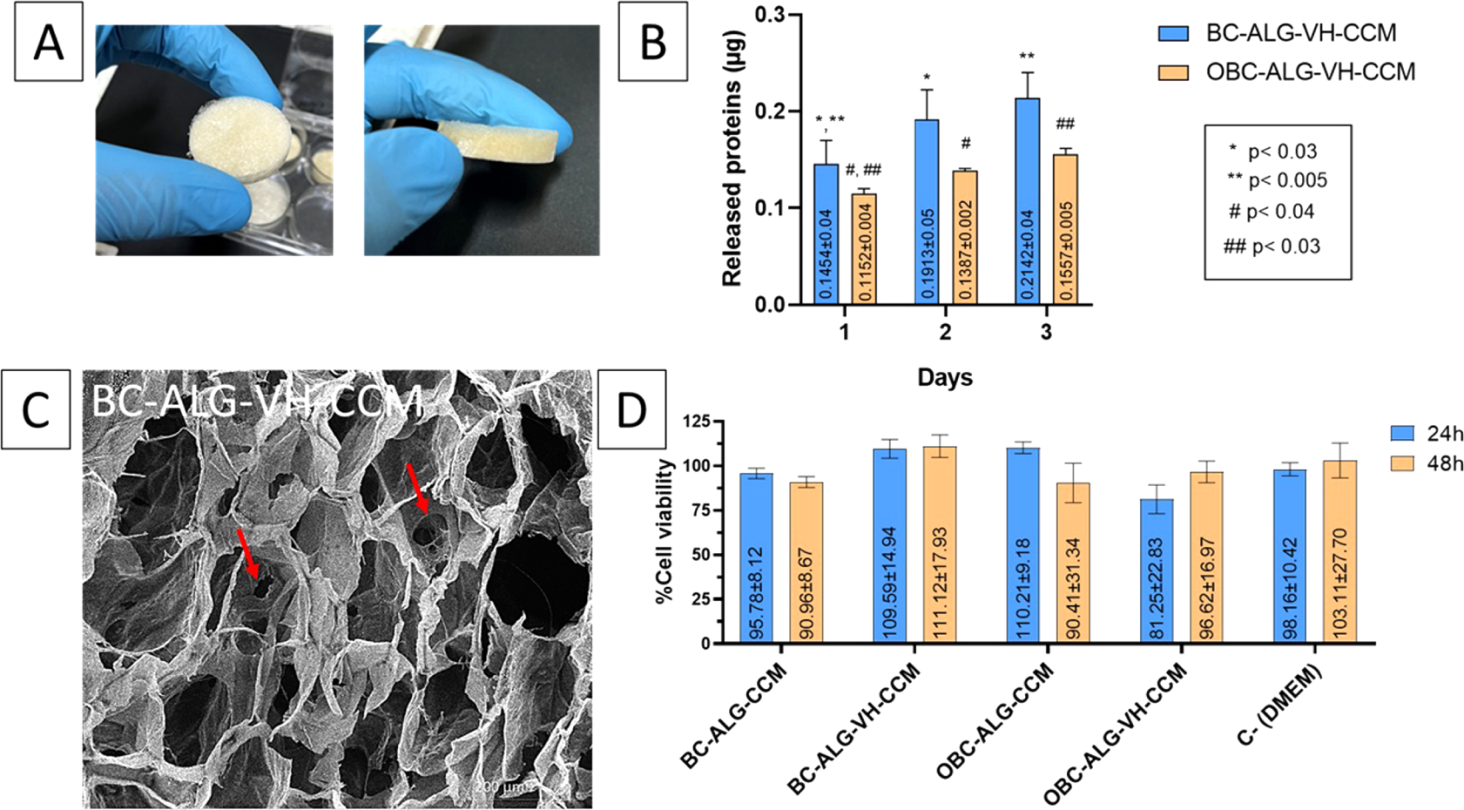
(A) Representative images of the membranes produced in this study, all exhibiting uniform and spongy morphology. (B) Cumulative protein release (both VH and CCM) from BC-ALG-VH-CCM (blue) and OBC-ALG-VH-CCM (orange) over 3 days in PBS, quantified using the Bradford assay. (C) Scanning electron microscopy (SEM) image of the BC-ALG-VH-MCC membrane, with red arrows showing pore connectivity. (D) Viability of keratinocytes (HaCaT) after 24 and 48 h of exposure to membrane extracts. Release proteins and cytotoxicity assays were conducted in triplicate (*n* = 3). Two-way ANOVA followed by Tukey’s post hoc test was used for statistical analysis. Asterisks (*) indicate statistically significant differences within the BC-ALG-VH-CCM group compared to day 1; hashes (#) indicate statistically significant differences within the OBC-ALG-VH-CCM group compared to day 1. Data are presented as mean ± SD represented by error bars (*n* = 3), analyzed by two-way ANOVA with Tukey’s post hoc test (*p* < 0.05). Data represent mean ± SD represented by error bars (*n* = 4); *p* < 0.05.

Figure 3B presents the cumulative protein release profiles of VH and CCM from two formulations: BC-ALG-VH-CCM (blue bars) and OBC-ALG-VH-CCM (orange bars), quantified using the Bradford assay. Both materials exhibited a characteristic burst release on day 1, followed by a gradual decline in cumulative release over the next 2 days. This behavior is typical of controlled-release systems, where initially adsorbed proteins are rapidly released into the medium, while residual protein diffuses more slowly through the matrix or is released via gradual degradation. The BC-based membrane released significantly more protein than the OBC-based membrane, possibly due to differences in matrix porosity or interaction with the bioactives. Additional factors may also contribute to this difference. For instance, oxidation of BC introduces carboxyl groups that could enhance electrostatic interactions with protein molecules, thereby retaining proteins more strongly within the OBC matrix. Although only cumulative release was assessed, the observed pattern suggests an initial high-dose phase followed by sustained release, which can be advantageous for therapeutic applications such as wound healing.

Figure 3C shows a scanning electron microscopy (SEM) image of the BC-ALG-VH-CCM membrane, with red arrows highlighting pore interconnectivity. This structural feature is important for tissue engineering applications, as interconnected pores facilitate oxygen and nutrient diffusion, as well as potential vascularization. SEM images of all formulations are presented separately in Figure 4, enabling comparative analysis.

**Figure 4:**
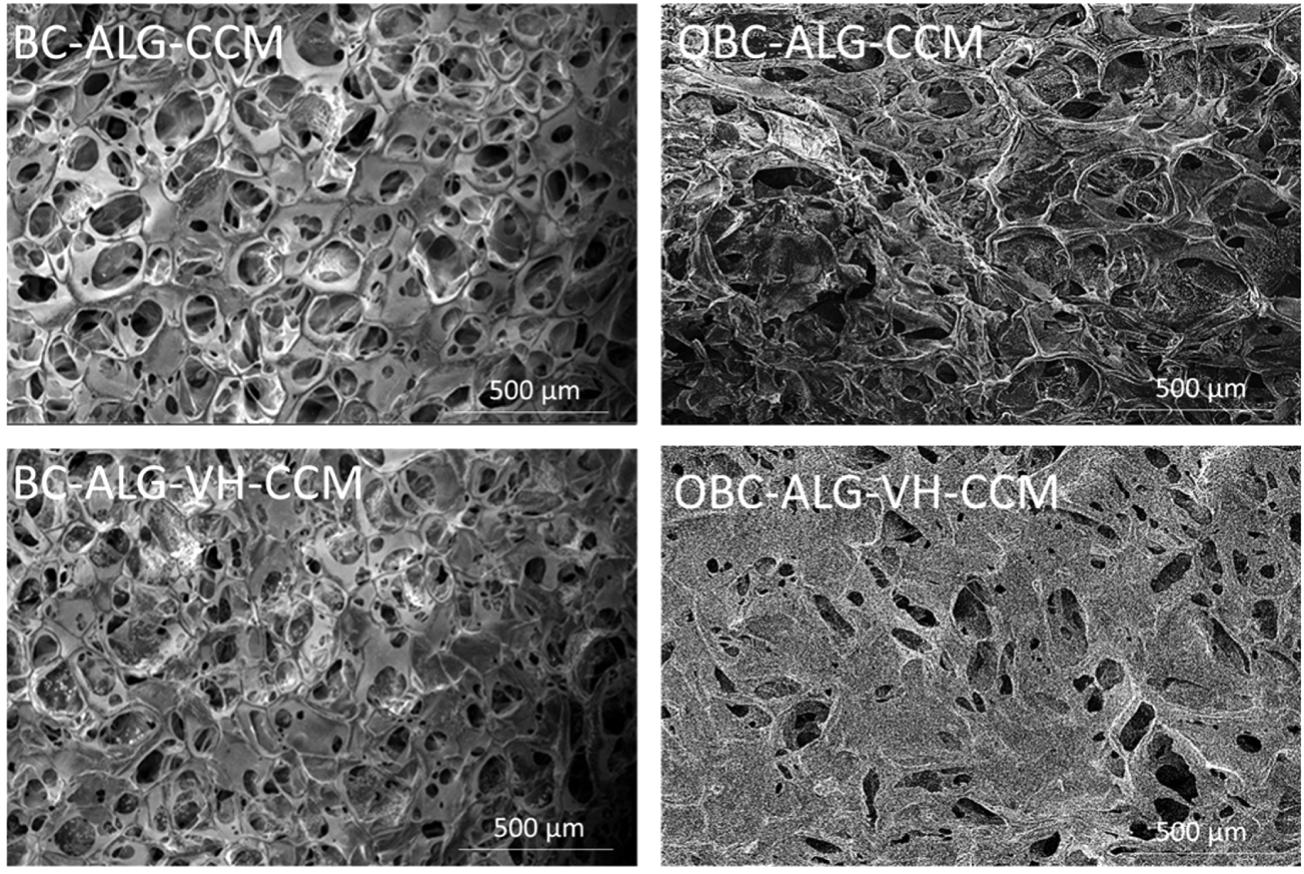
Scanning electron microscopy (SEM) images of the membranes: BC-ALG-CCM, OBC-ALG-CCM, BC-ALG-VH-CCM, and OBC-ALG-VH-CCM.

Figure 3D displays the results of keratinocyte viability after 24 and 48 h of exposure to membrane extracts. All membranes were noncytotoxic, with cell viability exceeding 80% in all conditions, which may reflect trophic or protective effects of the incorporated bioactives.

Scanning electron microscopy (SEM) was employed to examine the microstructure of the membranes in greater detail, providing high-resolution images of their surface morphology (Figure 4). All membranes produced exhibited pores on their surface, revealing differences between the samples based on native bacterial cellulose (BC) and oxidized BC (OBC). The pores in the oxidized matrices were smaller, which may be associated with a more efficient deposition of the active vitreous humor (VH) and CCM in these samples. Notably, the oxidized BC matrix contains functional groups that could enhance its interaction capacity, potentially improving the incorporation and stability of bioactive molecules within the material.

To assess the epithelial healing potential of the developed membranes an *in vitro* scratch assay was performed using L929 fibroblasts. This method is widely used to evaluate cell migration, proliferation, and regenerative effects of biomaterials and bioactive compounds.^34^ In our study, cells were treated with extracts of the different membrane formulations and observed over a 24 h period, with images captured every 6 h (Figure 5).

**Figure 5:**
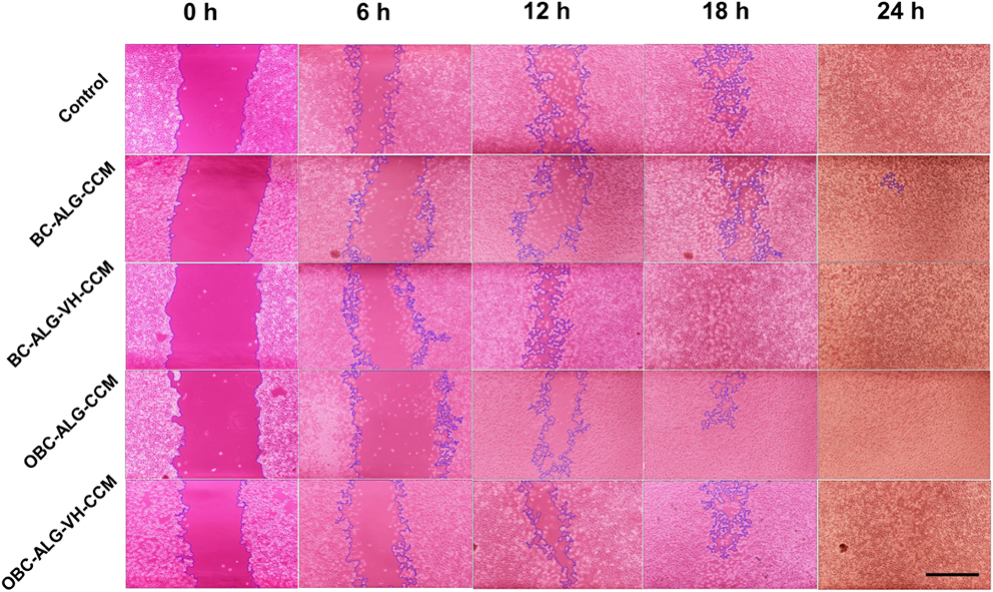
Photographs of the *in vitro* scratch assay against L929 fibroblasts treated with extracts of the developed membranes. Representative images of the regenerative/closing process of L929 cells treated with each sample are shown. The blue lines delineate the edges of the scratched areas (no cell layer inside them) that were detected by ImageJ software. All images are on the same scale, the black bar in the lower right corner is equivalent to 120 μm.

Similar to the control group, all samples achieved complete scratch closure after 24 h, but the BC-ALG-HV-CCM membrane presented better performance closing the scratch in 18 h. These results suggest the potential of the BC-ALG-HV-CCM membrane for applications in skin healing, and further *in vivo* studies are required to validate its effectiveness as a dressing. Figures 5 and 6 presents the graphs showing the scratch area closure and the area/time closure rates. According to the statistical analysis (Two-Way ANOVA), significant differences were observed in the scratch areas at the different time points for both tests.

**Figure 6:**
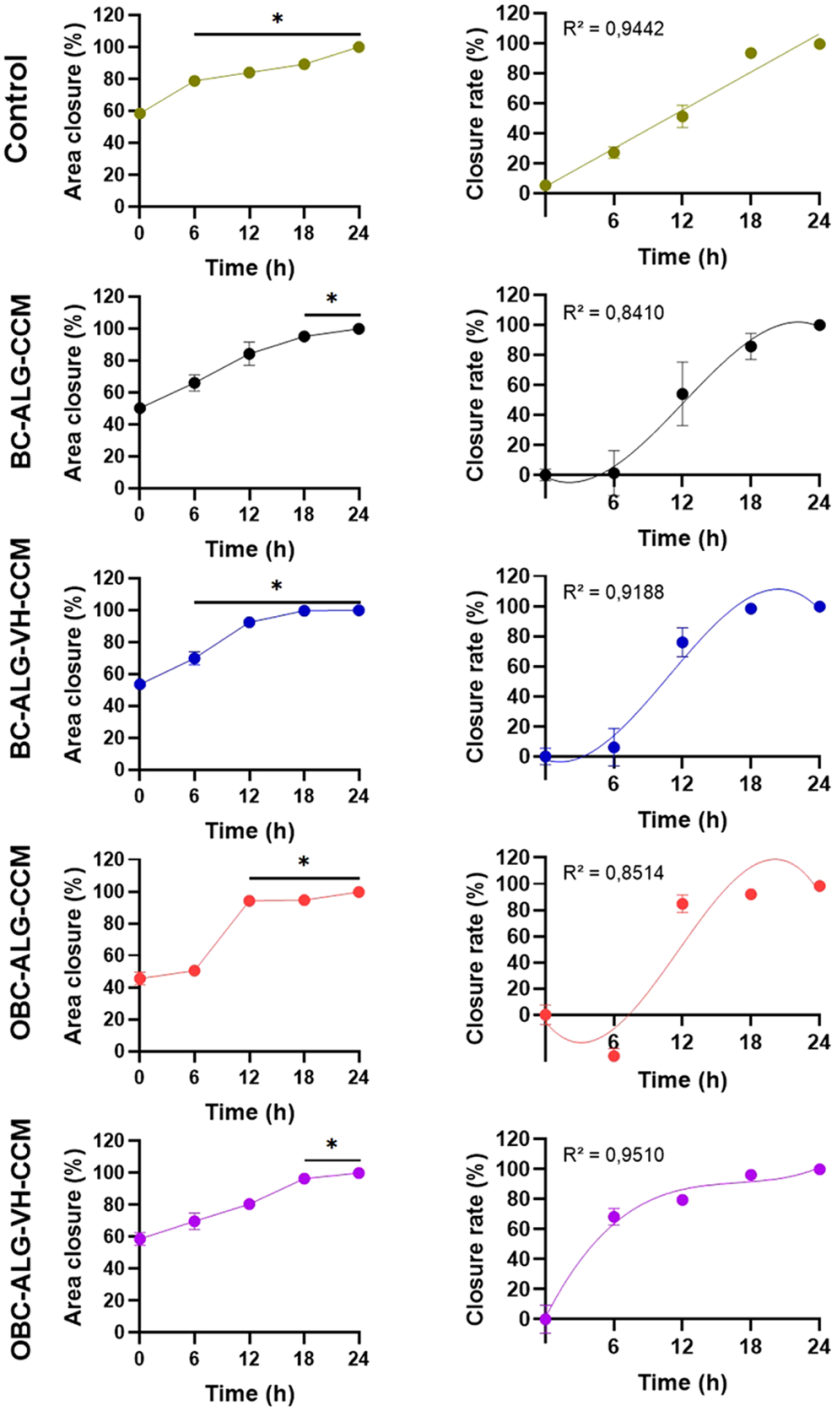
Area closure and closure rate of the cut of the *in vitro* healing test after treatment with the sample extract. Percentage values of the area closure and closure rate were obtained after treatment of L929 cells with membrane extracts and sectioning. Photographs of the sections were analyzed every 6 h up to 24 h. Linear and nonlinear regressions were performed on the closure data to determine the best fit. *p*-values < 0.05*. Some error bars are shorter than the size of the symbols.

Regarding the performance observed in the BC-ALG-CCM group, which presented a lower closure rate than the control group, we acknowledge that this finding may initially appear contradictory. However, we propose a few hypotheses that may help explain this behavior. The absence of the vitreous humor (VH) a component rich in structural proteins and extracellular matrix-mimicking bioactive signals may have limited the ability of the BC-ALG-CCM membrane to provide an optimal microenvironment for cell migration and proliferation. This suggests that the presence of CCM alone, although biologically active, may be insufficient to support efficient in vitro wound healing. Furthermore, the isolated release of CCM components without the complementary structural and functional cues provided by VH may have disrupted the cellular signaling balance, leading to suboptimal proliferative or migratory response. This interpretation is supported by the statistical behavior of this group.

On the other hand, the groups incorporating VH particularly BC-ALG-VH-CCM and OBC-ALG-VH-CCM demonstrated significantly improved in vitro wound closure, supporting the hypothesis of a synergistic interaction between VH and CCM. This effect likely arises from the combination of structural cues provided by VH proteins, which mimic the extracellular matrix, with paracrine biochemical signals derived from extracellular vesicles and soluble proteins present in CCM.

Regression analyses of the wound closure data (Figure 6) revealed that the cell proliferation/migration patterns for cells exposed to the membrane extracts fit a cubic regression model, while the control group followed a linear regression model. Among several models tested, the cubic model yielded the highest coefficient of determination (*R* ^2^), indicating its superior explanatory power. This divergence suggests that external components introduced by the membrane extracts, such as VH and CCM, altered cellular behavior compared to the baseline culture medium. However, we emphasize that it is not yet possible to determine whether VH or CCM has a greater individual effect, as both likely contribute through complementary mechanisms. This underscores the importance of future mechanistic studies aimed at dissecting their individual roles, including the profiling of cytokines, growth factors, and related signaling pathways. Finally, we acknowledge that the scratch assay, while informative, represents a simplified two-dimensional in vitro model. To confirm the therapeutic potential of these functionalized membranes for wound healing, in vivo studies are essential to evaluate their efficacy under more physiologically relevant conditions, including aspects such as immune response, vascularization, and tissue remodeling. These experiments are already being planned as part of our ongoing research efforts.

## 4 Conclusion

This study demonstrated that bacterial cellulose alginate membranes functionalized with bioactive compounds derived from unconventional sources specifically, vitreous humor proteins and conditioned medium from dental pulp stem cells possess a set of synergistic properties desirable for epithelial tissue regeneration. The membranes exhibited high swelling capacity and porosity, which is favorable for exudate control and gas exchange. They also had hydrophilic surfaces that promote interaction with biological fluids and the ability to release proteins in a sustained manner. *In vitro* assays confirmed that all formulations were noncytotoxic and supported keratinocyte viability, with the VH + CCM group showing a distinct influence on wound closure dynamics. These findings suggest that the combination of extracellular matrix-like cues (from VH) and paracrine signaling molecules (from CCM) may modulate cell behavior in a coordinated manner. Although further validation particularly through *in vivo* studies is necessary, the results presented here highlight a promising platform for developing sustainable, bioactive wound dressings. By integrating waste-derived biomolecules into functional materials, this work contributes to both regenerative medicine and circular bioeconomy strategies.
